# Intrinsic brain network dynamics modulated by neural stimulation to cerebellum

**DOI:** 10.1162/NETN.a.541

**Published:** 2026-04-22

**Authors:** Kanika Bansal, Zaira Cattaneo, Viola Oldrati, Chiara Ferrari, Emily D. Grossman, Javier O. Garcia

**Affiliations:** Humans in Complex Systems Division, U.S. Army DEVCOM Army Research Laboratory, Aberdeen Proving Ground, MD 21005 USA; Department of Computer Science and Electrical Engineering, University of Maryland, Baltimore County, MD USA; Department of Human and Social Sciences, University of Bergamo, Italy; Scientific Institute, IRCCS E. Medea, Bosisio Parini (LC) 23842, Italy; IRCCS Mondino Foundation, Pavia 27100, Italy; Department of Humanitas, University of Pavia, Pavia 27100, Italy; Department of Cognitive Sciences, University of California, Irvine, Irvine, CA USA

**Keywords:** Brain network reconfigurations, Cerebellum, rTMS, Flexibility, Integration

## Abstract

The cerebellum, with its distinctive architecture and extensive cortical connections, has long been recognized for its highly structured interconnectivity with the cortex and has been proposed as part of a larger circuit that shapes brain network dynamics. Here, we evaluate dynamic network reconfigurations in resting-state fMRI connectivity pre- and post-noninvasive inhibitory repetitive transcranial magnetic stimulation targeting the right Crus I of the cerebellum. Using dynamic community detection to evaluate the stimulation’s effect on modular network structures, we characterize the network properties by which cerebellar stimulation spreads through the cortex. We find that: (a) the *flexibility*, or the likelihood of network nodes to change module allegiances, increased post stimulation; (b) the dynamic patterns by which module allegiances emerged and evolved were highly individual and did not follow a single functional prototype; and (c) the cerebellar nodes had connectivity properties of integrators for distinct network modules. These results are consistent with the idea that cerebellum is pivotal in modulating distributed cortical activity by restructuring the integration and segregation of neural networks. This integrative capacity of the cerebellum may underlie its proposed role in coordinating neural systems, including those supporting higher cognitive function.

## Author Summary

The cerebellum, with its distinctive structure and widespread cortical connections, plays a central role in cortical network dynamics that support higher cognition. Here, using resting-state fMRI connectivity, we evaluate how inhibitory repetitive transcranial magnetic stimulation to the right Crus I of the cerebellum alters dynamic network reconfigurations across the cortex. Dynamic community detection revealed three main effects: (a) network flexibility, the tendency of nodes to switch module affiliations, increased after stimulation; (b) the temporal evolution of module allegiances varied strongly across individuals, lacking a common functional prototype; and (c) cerebellar nodes acted as integrators linking distinct network modules. These results highlight the cerebellum’s pivotal role in reshaping cortical integration and segregation, supporting its proposed function in coordinating distributed neural systems underlying cognition and adaptive behavior.

## INTRODUCTION

The neural underpinnings of cognition are rooted in the complex coordination of distributed neural systems that support unique functions. Often underrecognized is the role of the cerebellum in this process. Most clearly understood as supporting the coordination and timing of highly skilled motor control, the cerebellum is now recognized as a potent modulator of cognitive, language, social, and affective functions (Buckner, 2013; Cattaneo et al., 2022; Leiner et al., 1993; Schmahmann, 2010; Schmahmann et al., 2019). Cognitive impairments observed after cerebellar lesions—termed *dysmetria of thought—*manifest in the regulation of speed, capacity, and appropriateness of cognitive processes, especially in forming predictions that may improve the efficiency with which cognitive processes are executed (Honda et al., 2018; Van Overwalle et al., 2020). This disruption parallels the cerebellum’s role in mediating highly skilled motor behaviors through the fine-tuning of movement execution (Koziol et al., 2014); however, the means by which computational mechanisms in the cerebellum promote efficiency in cortical processing are not yet fully understood.

The cerebellum is organized as orderly topographic maps that connect to corresponding regions of the cerebral cortex, with all functional networks represented (Buckner et al., 2011; Diedrichsen et al., 2011). Importantly, each microfold of the cerebellum has an architecture that is highly uniform, organized as a closed loop circuit with a single cortical target (Leiner et al., 1993; Strick et al., 2009). This observation is the basis of the universal cerebellar transform theory (Schmahmann & Pandya, 1997), which posits that each subdivision of the cerebellum executes the same computation and it is the cortical target of the cortico-cerebellar loop that determines any functional specialization (Leiner et al., 1986; Schmahmann et al., 2019). It is important to note, however, that even with uniformity in circuitry, unique computations are likely to emerge across cognitive and motor subdivisions of the cerebellum as a direct result of both the input structure and the nature of the reinforcement signals, leading to multifunctionality (Diedrichsen et al., 2019).

More recent proposals implicate the cerebellum in shaping the functional dynamics of cortical networks. Evidence in favor of this role comes from inactivation studies in which inhibition delivered to the cerebellum disrupts the relative phase of ongoing local neural oscillations between distal cortical sites, specifically disrupting the coherence of functionally connected circuits (Georgescu Margarint et al., 2020; McAfee et al., 2022). This empirical evidence is consistent with recent theoretical proposals in which the cerebellum is a key component in thalamocortical circuits that regulate and stabilize temporal dynamics in cortical network states (Heck et al., 2023; Shine, 2021). In these proposals, cerebellar inputs to the thalamus promote segregated processing that is the hallmark of distinct cognitive functions, and a proposed mechanism by which efference copies support rapid anticipation of ongoing sensory events. Impairments to the cerebellum, therefore, disrupt this mechanism of constraining cortical dynamics and create the potential for less constrained and more integrative network states. Comodulating the coherence of functionally connected targets and shaping the landscape of cortical dynamics are key mechanisms by which the cerebellum has the potential to promote the efficiency of well-learned spatiotemporal patterns.

Neurostimulation approaches, such as transcranial magnetic stimulation (TMS) and transcranial electrical stimulation, have proven instrumental for revealing the connectivity dynamics of cortico-cortical and cerebellar-cortical interactions. These methods enable reversible modulation of the underlying local neural activity that propagates to distal, functionally connected circuits, uncovering causal relationships within large-scale cognitive systems (Garcia et al., 2011). When applied to the cerebellum, neuromodulation has demonstrated potential to perturb connectivity of cortical targets. For example, inhibitory theta burst stimulation over Crus I, a cognitive hub in the lateral cerebellum, induced a reduction of connectivity with frontal control regions in prefrontal cortex (Rastogi et al., 2017). Likewise, excitatory neuromodulation delivered to the same target increases connectivity to the cortical nodes of the default mode network, distinct from the impact of midline vermis stimulation that instead increased connectivity to the dorsal attention network (Esterman et al., 2017; Halko et al., 2014).

Advancements in network neuroscience, such as dynamic community detection, characterize the reorganization of neural communities over time, at both local and global scales (e.g., (Garcia, Ashourvan, et al., 2020; Garcia, Battelli, et al., 2020; Garcia et al., 2018)). This suite of tools and those belonging to the more general *network neuroscience* have demonstrated the cerebellum to have small-world properties that, similar to the cerebral cortex, balances regular connectedness with random connectivity (Chen et al., 2022). Together, these approaches provide an integrated strategy by which the intrinsic cerebro-cerebellar connectivity may promote cohesion in cortical community structure.

In this study, we use an intervention approach to characterize the neuromodulatory role of the cerebellum on cortical targets, specifically evaluating the hypothesis that cognitive subdivisions of the cerebellum function to stabilize cortico-cortico functional network structure. To achieve this, we compute the dynamic network properties of cortical communities (or network modules) in the resting state, employing a “perturb and measure” approach to noninvasive cerebellar stimulation. Specifically, we compare network dynamics in resting-state fMRI before and after administering 1-Hz inhibitory repetitive transcranial magnetic stimulation (rTMS) to the right cerebellum Crus I region, including shifts in the node dynamics and transient impacts on the overarching community structure, emphasizing the integrative and recruitment properties of the networks. Finally, we evaluate the dynamic hub and integrative properties of the cerebellum relative to cortical nodes to assess how well positioned cerebellar targets are to modify downstream network structure.

## METHODS

### Experimental Data Acquisition

Nineteen participants that met the inclusion criteria for participation in both the TMS and MRI procedures were recruited for this experiment. Two participants were excluded from the analysis because they did not participate in all experimental sessions. Hence, 17 individuals (3 males; *M* = 25.6 *SD* = 1.9 years old) were included in the final sample. All subjects provided informed consent as approved by the Ethical Committee of the Istituto di Ricovero e Cura a Carattere Scientifico Mondino Foundation (Pavia, Italy).

#### Procedure.

Participants completed three experimental sessions on three separate days. Each session for each participant was scheduled at the same time of the day; each consecutive session was interleaved by a minimum of 2 days to a maximum of 15 days. In session 1, participants participated in a single-pulse stimulation to assess their resting-state motor threshold. In session 2, participants underwent two consecutive resting-state scans, during which T1 and T2*-weighted images were acquired. In session 3, participants completed two resting-state scans, one pre and one post the offline inhibitory rTMS protocol.

#### Stimulation.

In session 1, TMS was administered using a MagProx100 stimulator (MagVenture) connected to a butterfly-shaped coil, with static cooling (MCF-B70 coil, MagVenture). Single-pulse TMS was applied over the left primary motor cortex (M1) at increasing intensities to determine each participant’s resting motor threshold (rMT). The rMT was defined as the minimum stimulation intensity required to elicit motor-evoked potentials with an amplitude of at least 50 mV in the first dorsal interosseous muscle with a 50% probability (Rossini et al., 1994). In session 3, participants received 1-Hz rTMS for 20 min over the right cerebellum Crus I (Talairach coordinate 22, −75, −21) at 100% rMT as assessed in session 1 and in line with previous TMS studies targeting the cerebellum (Demirtas-Tatlidede et al., 2011; Ferrari et al., 2018). The cerebellar target region was identified by neuro-navigated TMS (using the neuronavigation system SofTaxic, EMS srl, Italy) on each participant’s individual T1-weighted anatomical images acquired in session 2. Offline inhibitory stimulation was delivered using a refrigerated coil connected to the stimulator, and the rTMS coil was positioned tangentially to the scalp, parallel to the midsagittal line, with the handle pointing superiorly, following evidence that this orientation effectively modulates cerebellar activity (Bijsterbosch et al., 2012; van Dun et al., 2017).

#### Image acquisition.

MR images were acquired using a 3 T (Philips Healthcare, Best, The Netherlands) scanner with a 32-channel head coil. In session 2, T1-weighted images were collected for each individual (192 images, TR = 10 s, TE = 101 ms). Subjects then participated in a single 18 min 40 s resting-state scan, with the instructions to lay quietly with eyes open during which T2*-weighted images were collected (3 × 3 × 3 mm voxels with .3-mm gap, 560 volumes, TR = 2 s, TE = 27 ms). In session 3, participants repeated the resting-state scan two additional times, once prior to and once immediately following the rTMS stimulation. Poststimulation fMRI image acquisition was initiated within *M* = 2.8, *SD* = 0.49 min (range: 2.5–4 min) after cessation of the stimulation.

#### Data preprocessing.

All functional images were preprocessing in BrainVoyager (Brain Innovations, Inc.), including corrected for head motion within the scan, spatial smoothing (4-mm Full Width at Half Maximum gaussian kernel), linear detrending, and temporal high-pass filtering (cutoff frequency: 0.01 Hz). Functional images were then registered to the individual subject volumetric T1-weighted anatomical images. Cortical regions of interest (ROIs) were identified using the Schaefer template (Schaefer et al., 2018) (resolution: 200 parcels per hemisphere) applied using *mris_ca_train* to the native white/gray boundary of the cortical surface (smoothwm) as generated using Freesurfer’s *recon-all*. Cerebellar ROIs were identified using the Diedrichson probabilistic atlas of the human cerebellum (Diedrichsen et al., 2011, 2009), normalized to MNI152 atlas space. Vertex time courses were extracted using custom MATLAB (MathWorks, Inc.) scripts in conjunction with Neuroelf (https://neuroelf.net/). Time courses *z*-score normalized then averaged into a single time series per parcel.

### Connectivity Estimation

Functional connectivity was estimated using coherence measures between different ROIs in the cerebral and cerebellar cortices. Coherence measures were computed using the wavelet toolbox (Grinsted et al., 2004) in MATLAB (MathWorks, Inc.). We optimized the temporal window and frequency range of time-resolved functional connectivity using wavelet coherence to optimally capture task-related associations (Telesford et al., 2016) within functionally meaningful fluctuations in the BOLD signal (Rissman et al., 2004), within the constraints set by the imaging technique and the underlying hemodynamic response function (HRF; Kuo et al., 2011). We estimated coherence within the 0.06- to 0.12-Hz band (HRF-constrained low-frequency range per Sun et al., 2004), applying a 40-s (20 TR) sliding window. This calculation yielded 28 unique and temporally sequential coherence matrices representing the strength of each pairwise connection across the atlas parcellation.

Sun et al. (2004) demonstrated that coherence analyses are most robust in the 0.06- to 0.12-Hz band, and Sun et al. (2005) further validated phase-derived coherency for capturing temporal network properties. Later studies (e.g., Bassett et al., 2011; Ekman et al., 2012) adopted this range to study dynamic network flexibility in cognitive and motor tasks. A 20-frame (40 s) sliding window was chosen to account for both the temporal resolution imposed by the TR (2 s) and the low-pass filtering property of the HRF. This window length provides ∼2.4 cycles at 0.06 Hz and ∼4.8 cycles at 0.12 Hz, sufficient for stable coherence estimation while preserving sensitivity to network reconfiguration. Window size does affect results: Shorter windows capture faster fluctuations but increase variance, whereas longer windows reduce variance but obscure transient states (Telesford et al., 2016). Thus, the 20-frame window represents a principled compromise that optimizes both spectral sufficiency and temporal sensitivity for detecting dynamic changes in brain networks.

### Dynamic Community Detection

**Figure 1.**
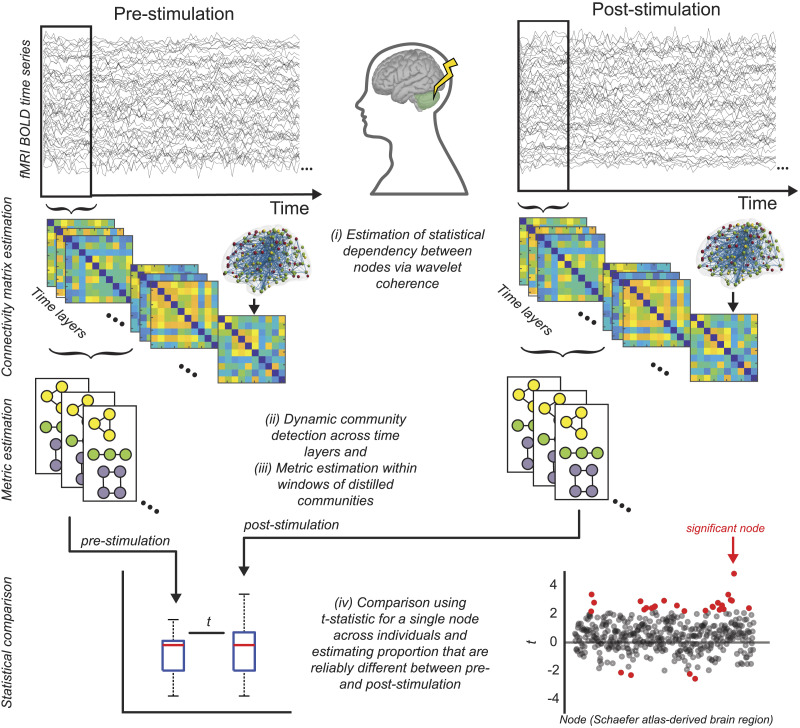
Schematic of the experimental and analysis design. Resting-state fMRI activity was measured before and immediately following 20 min of 1-Hz inhibitory active stimulation (rTMS) delivered to the right cerebellum. Our analysis steps included: (a) the estimation of functional connectivity between 432 ROIs (400 cortical parcels spanning left and right hemispheres, and 32 cerebellar ROIs) via wavelet coherence in nonoverlapping temporal windows (40 s each); (b) dynamic community detection on the temporal connectivity matrices (time layers) to distill a series of community structures; (c) extraction of complex network metrics quantifying network reconfigurations; and (d) statistical comparisons using the *prestimulation* condition as the baseline.

We employed dynamic community detection algorithms across 28 temporal layers of functional brain connectivity, to evaluate the modular organization of the brain before and after cerebellar stimulation. The algorithm optimizes a modularity function Q to distill complex connectivity matrices into a series of coarse clusters or communities of networks across time and was implemented using generalized Louvain algorithm and standard optimization procedures (for a review, see Garcia et al., 2018; Lima Dias Pinto et al., 2024; Mucha et al., 2010).

$$Q_{G}=\frac{1}{2\mu }\sum_{\text{ijlr}}\left[\left(A_{ijl}-\gamma \frac{k_{il}k_{jl}}{2m_{l}}\right)\delta_{lr}+\delta_{ij}\omega \right]\delta \left(C_{il},C_{jr}\right),$$ (1)

where *Q*_*G*_ is the generalized multilayer modularity function, indices *l* and *r* denote consecutive time layers (as shown in Figure 1), *A*_*ijl*_ is the weighted edge between nodes *i* and *j* in layer *l, k*_*il*_ is the degree of node *i* in layer *l, m*_*l*_ is the sum of the edge weights of layer *l, μ* is the sum of the edge weights of all time layers, *C*_*il*_ is the community affiliation of node *i* in layer *l*, and *δ* is the Kronecker delta, which equals 1 if *i* = *j*, and 0 otherwise. The community assignments are dependent on two parameters: (a) a structural resolution γ parameter and (b) a temporal resolution ω parameter. We used *γ* = 1 and *ω* = 1 in this analysis. This, on an average, yielded four communities (*SD* = 0.22, Supporting Information Figure S1) that spanned the 18 min 40 s scan for both the pre- and postcerebellar stimulation conditions. Due to the heuristic nature of the generalized Louvain algorithm, we ran 100 iterations of community detection for every individual and condition (pre- and poststimulation) and the estimated metrics (discussed below) were averaged across these iterations.

### Quantifying Dynamic Reconfigurations

In our study, we computed a range of network metrics to quantify the dynamic reconfigurations in the cerebral cortex and cerebellar connectivity following cerebellar stimulation. Our metrics of interest fall into two broad categories. The first category includes metrics that are agnostic to any reference community structure and estimate nodal dynamic changes in affiliation: flexibility and promiscuity. The second category comprises metrics that are calculated relative to a reference community structure (see the Consensus Community section below): integration, recruitment, participation coefficient, and within-module degree. Together, these metrics provide a comprehensive view of the dynamic community structure and the roles individual nodes play within this structure.

#### Node allegiance.

We calculated allegiance matrices to assess the pairwise relationship of the brain regions. Allegiance accounts for the proportion of the total time a pair of nodes belongs to the same community, and is defined as:

$${\text{Allegiance}}_{jk}=\frac{1}{L}\;\sum_{t=1}^{L}\delta_{C_{j}\left(t\right)C_{k}\left(t\right)},$$ (2)

Here *L* is the total number of time layers, *C*_*l*_(*t*) denotes the community that contains the node *l* at time *t*, and *δ* denotes the Kronecker delta such that *δ*_*C*_*j*_(*t*)*C*_*k*_(*t*)_equals 1 if the nodes *j* and *k* are in the same community at time layer *t* and equals 0 otherwise.

#### Flexibility and Promiscuity.

Flexibility of a node is estimated by calculating the number of times a node changes its community allegiance normalized by the total number of allegiance changes possible (Bassett et al., 2011). If *g*_*i*_ is the total number of times node *i* changes its affiliation and *L* is the total number of time layers, the flexibility of node *i* is

$$\xi_{i}=\frac{g_{i}}{L-1},$$ (3)

The promiscuity of a node is calculated as the fraction of all the communities in the network in which a node participated at least once (Papadopoulos et al., 2016). If a node participated in all the communities, its promiscuity score is 1.

#### Integration and recruitment.

These metrics provide insight into the structure of observed network reconfigurations in relation to a reference network structure. The integration coefficient of a node (or ROI) corresponds to the average probability that this node shares allegiance*—*either transiently or sustained*—*with nodes from other reference communities (Bassett et al., 2015; Mattar et al., 2015). The recruitment coefficient of a node corresponds to the average probability that this region is in the same network community as other regions from its own reference community (Bassett et al., 2015; Mattar et al., 2015).

#### Participation coefficient and within-module degree.

These metrics provide nuanced insights into the roles individual nodes play within their own communities and in the network at large. The participation coefficient helps in understanding a node’s role in intermodular connectivity, while the within-module degree focuses on intramodular connectivity. Here, we estimated these metrics using allegiance matrices to directly assess community-based reconfigurations.

The participation coefficient is a metric that quantifies the diversity of a node’s connections across different modules or reference communities in a network (Guimerà & Nunes Amaral, 2005). For a node *i*, participation coefficient *P*_*i*_ is calculated as:

$$P_{i}=1-\sum_{s=1}^{N}{\left(\frac{k_{\text{is}}}{k_{i}}\right)}^{2},$$ (4)

where *k*_*is*_ is the number of connections node *i* has to the nodes in community *s, k*_*i*_ is the total degree of node *i*, and *N* is the number of communities. The participation coefficient ranges from 0 to 1 such that a value close to 0 indicates that the node’s connections are primarily within its own reference community, while a value close to 1 suggests that the node has more uniformly distributed connections across multiple communities.

The within-module degree, often denoted as *z*, measures how well a node is connected within its own community or module. It is also calculated relative to a reference community structure and is defined as:

$$z_{i}=\frac{k_{i}-<k_{s_{i}}>}{\sigma_{k_{s_{i}}}},$$ (5)

here, *k*_*i*_ is the number of links of node *i* to other nodes in its module *s*_*i*_, <*k_s_i__* > is the average of *k* over all the nodes in *s*_*i*_, and *σ_k_s_i___* is the standard deviation of *k* in *s*_*i*_. A high *z* value indicates that the node is highly connected within its own community, serving as a potential hub or central node.

#### Dynamic hubs and dynamic integrators.

The nodes with high within-module degree act as dynamic hubs, and we defined them relatively to be in the top 5% of within-module degree. The nodes with low within-module degree but high participation coefficients particularly facilitate between-module connectivity. These nodes, defined as dynamic integrators, are in the lower 50% for within-module degree and top 50% for the participation coefficient.

#### Temporal variation in the metrics of interest.

We computed the metrics of interest on resting-state images collected pre- and postcerebellar stimulation. Within a condition, using a sliding window approach with 90% overlap between adjacent estimates, we obtained community structure for 10 consecutive windows or temporal layers to estimate the metrics of interest, which led to 19 estimation points for the entire scan, with each estimation point representing roughly 7 min of dynamics. This approach not only quantified the network reconfigurations but also allowed us to probe where the maximum effect of stimulation lies and how it changes immediately after stimulation due to underlying neurological processes.

### Consensus Community

Consensus communities in dynamic community detection refer to a technique that aims to identify stable or recurring patterns of community structure across multiple temporal windows or across different individuals. This is achieved by aggregating the community affiliations of nodes over time such that the modularity function across multiple layers (temporal or individual) is optimized to best represent the aggregated data. By focusing on consensus communities, the technique filters out transient or spurious community affiliations, thereby increasing the reliability of the detected community structure.

In this study, we obtained a consensus community structure for each individual (Supporting Information Figure S2) by first calculating a consensus similarity across all the 28 temporal windows for the total duration of each scan. Consensus similarity estimates a single representative partition from a set of partitions (here 28) that is the most similar to all others. We then calculated the consensus iterative for all the iterations (i.e., 100 iterations) of dynamic community detection. Consensus iterative identifies a single representative partition from a set of partitions (here 100), based on statistical testing in comparison to a null model (Bassett et al., 2013).

Individually obtained consensus communities allow for the exploration of individual differences in community structure, which can be essential for understanding subject-specific responses or conditions. It allows for more nuanced intersubject comparisons and can help in identifying subject-specific markers or predictors.

### Statistical Analysis and Dissimilarity Ratio

We estimated the metrics of interest for pre- and poststimulation conditions independently and used paired *t* tests to draw statistical comparison of various metrics between conditions. We report the dissimilarity ratio, which is the proportion of nodes that produced a significant difference (*p* < 0.05) within that region. To account for potential individual variability and test the 95% confidence interval of dissimilarity ratio, we also extracted a distribution of dissimilarity ratio after bootstrapping individuals that were included in *t*-test comparisons (picking 12 out of 17 individuals across all possible iterations). Mean effect size was estimated using the average values of Cohen’s *d* for the nodes that showed significant difference pre- and poststimulation (*t*-test *p* < 0.05). In addition to comparing the dissimilarity ratio, we also investigated the direction of differences (*t*-value magnitude) pre- and poststimulation. We computed a mean *t* value for the whole brain and compared it with a null model to assess how the observed change poststimulation compares to a change observed by chance (after scrambling the time series). To generate a null model, we performed *t*-test comparisons for each node after scrambling the BOLD time series and computing the mean *t* value; this process was repeated for hundred iterations to generate a null distribution. The null model produced low average *t* values indicating the absence of strong directional effect.

## RESULTS

Through a series of network dynamics analyses, we evaluated the overarching community structure, the dissimilarity ratio in network nodal properties before and after stimulation, and the nature of the dynamic network changes following active stimulation, focusing on the roles of integration and recruitment. Additionally, we characterized the dynamic hub and integrator nodes throughout the cortex based on metrics like the participation coefficient and within-module degree. As described in Figure 1, our analysis scheme involved five steps: (1) atlas parcellation of the cerebral and cerebellar cortices; (2) functional connectivity estimation via wavelet coherence in nonoverlapping sequential temporal windows; (3) dynamic community detection, distilling these functional connectivity matrices into a series of discrete communities; (4) dynamic metric calculation; and (5) statistical comparisons using the *prestimulation* condition as the baseline.

### Time-Evolving Community Structure

Given time-evolving network connectivity, particularly important for time-dependent effects of repetitive stimulation, we first identified the community structure in networks during the entire scan using dynamic community detection (Garcia et al., 2018; Lima Dias Pinto et al., 2024; Mucha et al., 2010). We leveraged modularity maximization, which aims to identify network communities or network modules such that within-community connections are maximized and between-community connections are minimized. Our analyses revealed a broad community structure that, on an average, consisted of four (*SD* = 0.22) communities within an individual for any given time window (Supporting Information Figure S1). This structure serves as the foundation for our subsequent analyses, providing a framework by which to understand the complex interplay between different regions of the brain.

### Dissimilarity Ratio Before and After Stimulation

**Figure 2.**
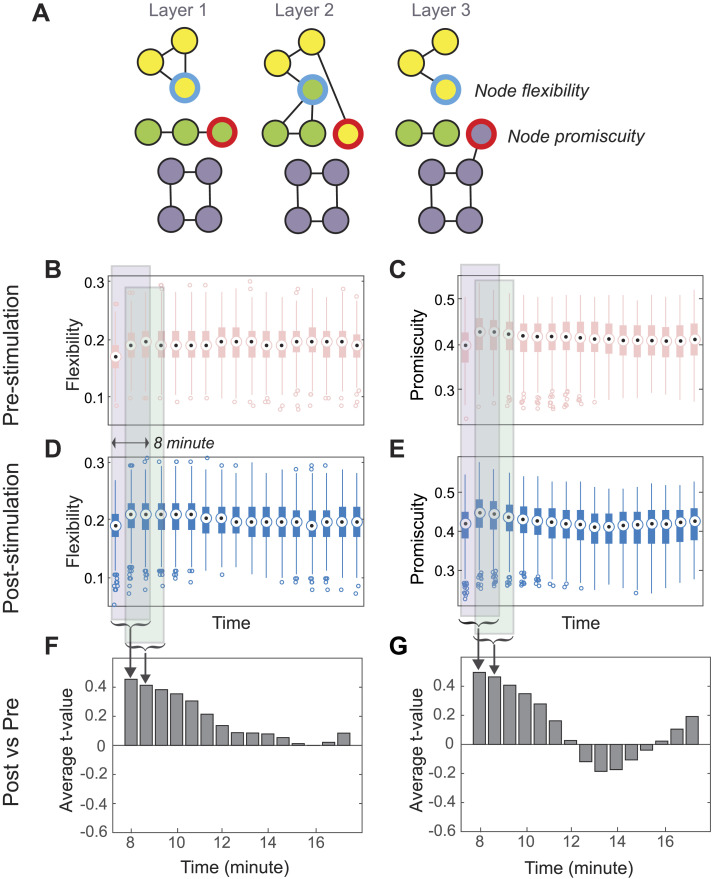
Impact of cerebellar stimulation on propensity of module or community reconfigurations. (A) We evaluated two nodal metrics, flexibility and promiscuity, which assess the likelihood and heterogeneity of nodal allegiance changes within the estimated communities or modules. Temporal variation of flexibility and promiscuity (B–C) pre-cerebellar stimulation and (D–E) poststimulation in resting state. (F–G) The effect of stimulation was computed using *t*-test comparisons between poststimulation and prestimulation conditions, over time, averaged for three consecutive windows, in a sliding manner. Here, we show the average *t*-score across nodes and it indicates the diminishing effect of stimulation over time with the first three windows capturing the immediate effects of the stimulation.

Figure 2 quantifies the dynamic community changes across time. *Flexibility* broadly refers to the likelihood that a node changes its community affiliation across time (Figure 2A). Increased flexibility has been linked to a variety of cognitive phenomena, including memory (Braun et al., 2015), learning (Bassett et al., 2011), persuasion (Cooper et al., 2018), and opinion change (Lima Dias Pinto et al., 2022), and has even been proposed to be a potential marker of cognitive flexibility (Xiao et al., 2022). As a complimentary metric to this generically dynamic metric, we also estimated *promiscuity*, which quantifies the proportion of communities that a node will affiliate with at least once through the time period of question. Whereas *flexibility* characterizes the coarse changes of a node across time, *promiscuity* gives sharper insight into the type of affiliative changes a node may traverse. To quantify the effect of cerebellar stimulation on network organization, we calculated these metrics in discrete windows across time for both pre- and poststimulation conditions (Figure 2B–E) and then performed a *t*-test comparison for each node. Average *t* values for the whole brain are depicted in Figure 2F–G as a function of time.

**Figure 3.**
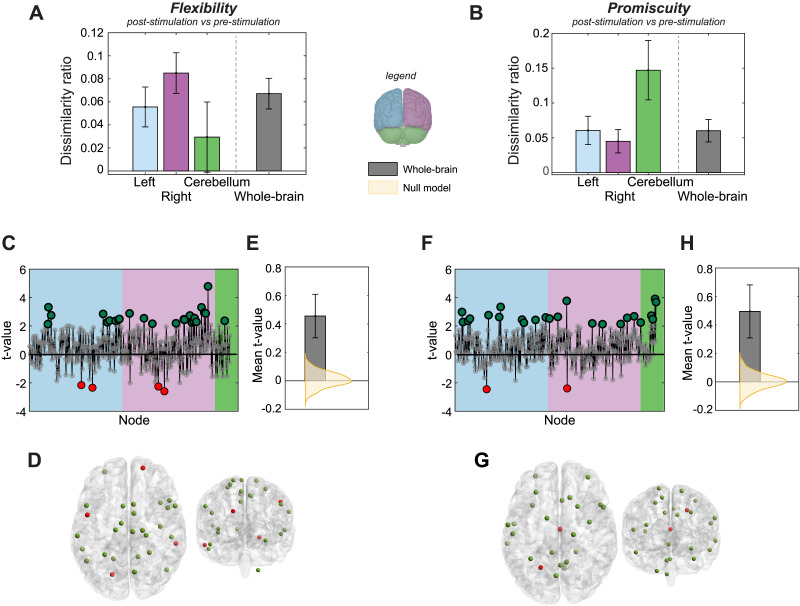
Flexibility and promiscuity increase post-cerebellar stimulation. (A–B) Dissimilarity ratio represents the proportion of nodes within each region (left and right cerebral cortices, cerebellar cortex, and whole brain) for which flexibility or promiscuity differed significantly between post- and prestimulation conditions (comparing metric distribution using a *t* test across individuals pre- and poststimulation for each node, *p* value < 0.05). Error bars represent the standard deviation of dissimilarity ratio after bootstrapping individuals that were included in *t*-test comparisons. (C and F) Estimated *t* values for each node when flexibility and promiscuity were compared between poststimulation and prestimulation conditions. Positive *t* values represent higher flexibility or promiscuity post stimulation. Larger dots represent those nodes with significant differences (*p* value < 0.05, uncorrected). (D and G) Distribution of nodes with significantly different flexibility or promiscuity post stimulation. Green nodes significantly increased metric dynamics post stimulation. (E and H) Average *t* value for the whole brain showing the strong directional effect of stimulation when compared with a null model generated after scrambling the temporal windows to lose pre- and poststimulation distinction. Error bar represents the standard deviation of the mean *t* value after bootstrapping individuals.

The “dissimilarity” ratio reflects the proportion of nodes that show significantly different values of the nodal metric (i.e., flexibility or promiscuity) before and after stimulation. We focused the dissimilarity analysis on the metrics derived from the first 8 min of imaging (240 volumes, three time windows) to understand the cortical effects of cerebellar stimulation, the interval over which the effects of stimulation were the most potent (also see Supporting Information Figure S2). This duration is consistent with the known (and observed, Figure 22F–G) decay of the effect of rTMS across time (Chen et al., 1997; Edwards et al., 2019; Pascual-Leone et al., 1998) and previous work that has shown network changes to dissipate within 15–20 min of rTMS in a similar protocol (Garcia, Battelli, et al., 2020; Muellbacher et al., 2000). As shown in Figure 3A–B, we computed the dissimilarity ratio separately for cerebral cortex and cerebellar cortex.

Inspection of the dissimilarity ratio across nodes of the cerebral cortex and cerebellum reveals an impact of rTMS on node dynamics for a small but significant proportion of the nodes. Notably, approximately 7% of nodes exhibited flexibility changes after rTMS (bootstrapping confidence interval [CI] = 3.9%–9%, mean effect size = 0.61), with a higher representation from the right cortical hemisphere as compared with the other regions. In contrast, the impact of rTMS on nodal promiscuity was concentrated in the cerebellum, where nearly 15% of the cerebellar nodes changed in promiscuity after stimulation (bootstrapping CI = 17.6%–5.9%, mean effect size = 1.01).

We next inspected the nature of the dynamic community changes, with Figure 3C and Figure 3F showing the direction of changes in flexibility and promiscuity across all nodes. The preponderance of nodes exhibited an increase in flexibility and promiscuity following cerebellar stimulation, both overall and among the nodes significantly impacted by stimulation. The spatial distribution of these stimulation-induced network dynamics (Figures 3D and 3G) revealed the nodes that significantly differed in their flexibility and promiscuity were not localized to a particular region of the brain; instead, they were distributed throughout the cerebral cortex, evidence for a more global response to cerebellar stimulation, at least cortically.

Figures 2 and 3 together demonstrate a strong brain-wide impact of cerebellar stimulation in network dynamics as revealed through dynamic flexibility and promiscuity. The overall average magnitude of dissimilarity, as revealed by individual node *t* values (Figures 3E and 3H), indicates strong directional effect when compared against a null model generated by scrambling the BOLD time series windows temporally, losing the distinction of before and after stimulation.

### Dissimilarity Ratio in Integration and Recruitment Due to Cerebellar Stimulation

**Figure 4.**
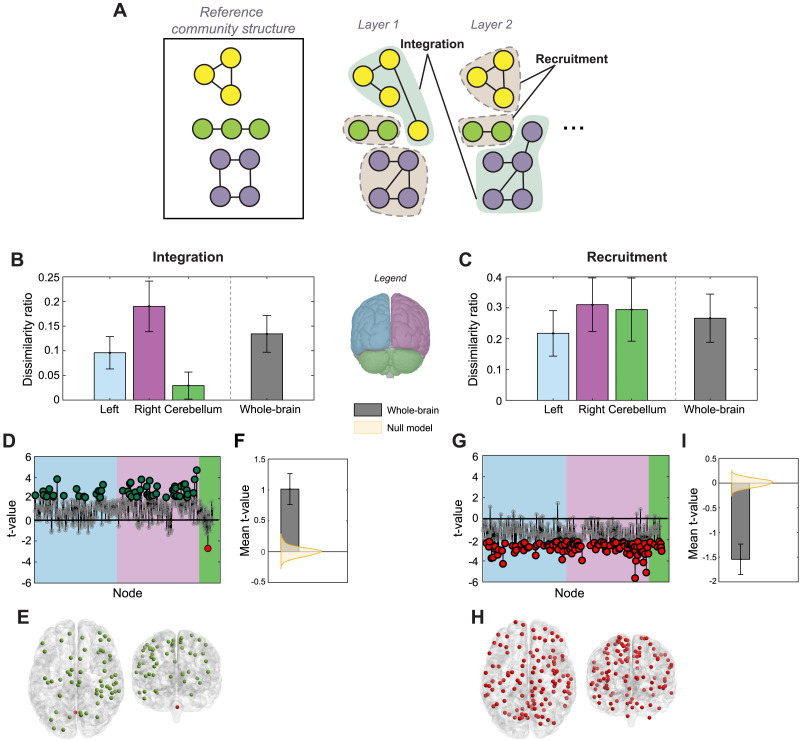
Community structure changes due to cerebellar stimulation. (A) Integration and recruitment quantify changes in node allegiances with respect to a reference community structure. Integration quantifies the probability of a node to be in the same community as the nodes from other reference communities, and recruitment quantifies the probability of a node to be in the same community as the nodes from its own reference community. (B–C) Dissimilarity represents the ratio of nodes within each region (left, right, and cerebellum, and whole-brain) for which integration or recruitment was significantly different between poststimulation and prestimulation conditions, using *t* tests (*p* value < 0.05, uncorrected). (D and G) Estimated *t* values. Positive *t* values represent higher integration or recruitment poststimulation. Larger dots represent significant differences. (E and H) Distribution of nodes with significantly different integration or recruitment post stimulation. Green implies an increase. Large proportions of nodes display significant changes in integration and recruitment, which we attribute to a stimulation-driven change in the overall community structure. (F and I) Mean of the *t* values for the whole brain, indicating strong directional effect of stimulation when compared with a null model generated by scrambling the temporal windows to lose pre and post stimulation distinction. Error bars in B, C, F, and I represent standard deviations calculated by bootstrapping individuals and using original, unscrambled temporal windows.

While promiscuity and flexibility can capture the changes of community affiliation across time, they do so in a very generic manner with no information on the specificity of these changes within the community structure. For this reason, we next inspected two other, reference-community-specific metrics that may capture systemic changes in network structure. Node *integration* refers to the average probability that a node shares community allegiance with nodes from other reference communities, reflecting intercommunity cohesion, while *recruitment* refers to the average probability that a node is in the same community as other nodes from its own reference community, reflecting within-community cohesion (Figure 4A).

We used the community structure in the prestimulation condition as a baseline modal community structure against which to compare changes due to cerebellar stimulation. We estimated the modal community structure across both time and subjects using a *consensus community* estimation (Bassett et al., 2013). As visualized in the consensus community structure in the resting-state data acquired prior to stimulation (Supporting Information Figure S3), we observed highly variable architectures when assessed across subjects. Thus, for these network metrics that are calculated relative to a *reference* community, we used the within-subject consensus community structure, as our data indicate this may best capture the dynamic changes of not only cerebellar stimulation but also best characterize the *resting-state* functional architectures within each individual (Supporting Information Figure S4).

We find that many cortical regions exhibited high levels of the dissimilarity from the consensus community structure following stimulation (Figure 4B–C). Approximately 20% of nodes in the right cortical hemisphere and approximately half of that (10%) in the left cortical hemisphere showed broad differences in integration following stimulation, with little change observed in the cerebellum nodes (right: bootstrapping CI = 26%–6%, mean effect size = 0.80 and left: bootstrapping CI = 15.2%–2.5%, mean effect size = 0.70). Conversely, a high proportion of nodes in both the right hemisphere and the cerebellum (~30%) showed changes in recruitment following stimulation, with slightly fewer in the left hemisphere (right: CI = 40%–7.5%, mean effect size = −0.86; left: CI = 33%–5.6%, mean effect size = −0.85, and cerebellum: CI = 41%–2.9%, mean effect size = −0.83).

As before, we next inspected the direction of change in integration and recruitment following rTMS (Figure 4D–E and Figure 4G–H). Interestingly, cortical nodes with significant changes in integration scores overwhelmingly showed an increase in integrative dynamics, evidence that neuromodulatory effects in cortical dynamics were distributed across networks. Likewise, all nodes with significant changes in recruitment scores following stimulation had lower scores of network recruitment, further evidence that neuromodulation over the cerebellum induced a fractionation in the existing community structure. We confirmed the robustness of this strong directional effect in both integration and recruitment by comparing the mean *t* value across all the nodes against a null model simulating no effect of rTMS, which was constructed by scrambling the pre- and poststimulation labels on the allegiance matrices (shown in Figures 4F and 4I).

### Dynamic Characteristics of the Cortical and Cerebellar Nodes

Finally, to understand the impact of cerebellar stimulation, we assessed the network properties of the cerebellar nodes by analyzing the *participation coefficient* and *within-module degree* in baseline (prestimulation) condition and comparing them to nodes in the cerebral cortex. In brief, the participation coefficient estimates the distribution of the edges (allegiances), with a high value suggesting distributed connections within different communities. This approach is particularly useful when estimated on allegiances, with some arguing that participation coefficient is a more meaningful measure for identifying truly integrative nodes as compared with degree-based approaches (Power et al., 2013). Within-module degree on the other hand quantifies how connected a node is to its own reference community. Thus, these two metrics inform whether nodes may be “dynamic hubs” (high within-module degree) or “dynamic integrators” (high participation coefficient and low within-module degree).

Note that this analysis is analogous to previous examinations of hubs and integrators using these metrics (e.g., Bertolero et al., 2018; Guimerà & Nunes Amaral, 2005; Power et al., 2013), with two exceptions: First, rather than focusing on direct functional connectivity estimates (i.e., wavelet coherence), our analyses were defined on *allegiance*, the pairwise estimate that a node-pair appears in the same community across time. We therefore estimated these metrics on dynamic network measurements rather than static. Dynamic network measurements also capture the evolving connectivity patterns, revealing regions that act as integrators by flexibly connecting with various communities or modules across different time points that cannot be revealed using static network measurements. Second, instead of using a predefined functional system as reference communities, we utilized individualized *consensus community* structures to calculate the network metrics allowing the *functional* networks to change based on the natural neural communication patterns within an individual. This individualized dynamic perspective highlights the node’s role in coordinating and integrating information across the brain, adapting to the shifting demands of cognitive and motor tasks for each individual.

**Figure 5.**
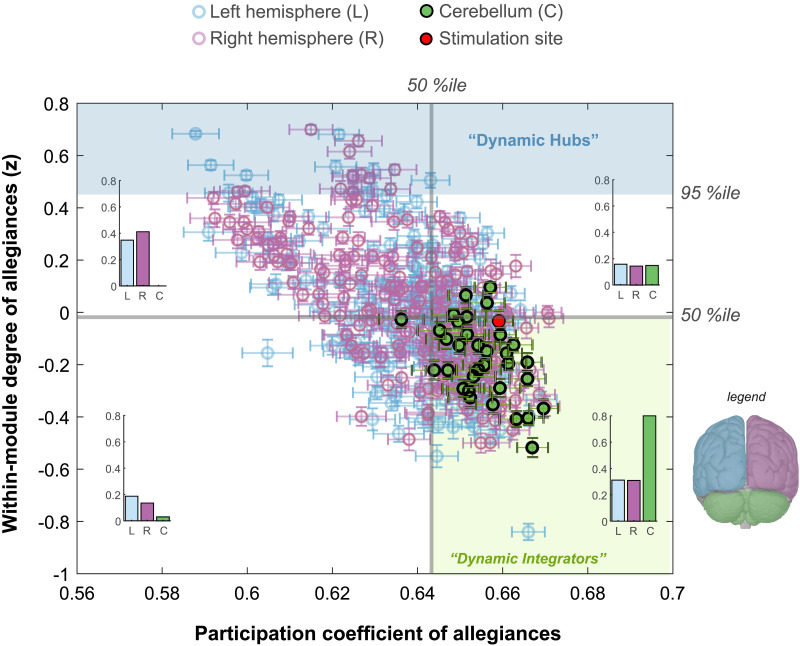
Dynamic characteristics of cortical and cerebellar nodes. Participation coefficient and within-module degree estimated using allegiance matrices for the baseline (prestimulation) condition. Here, the participation coefficient denotes the likelihood for a node to have allegiances with nodes outside of its reference community, and the within-module degree represents the tendency of a node to have allegiances with the nodes from its own reference community. Nodes with very high within-module degree (above 95%ile) are identified as dynamic hubs. None of the cerebellar nodes fall into this category. Nodes with high participation coefficient (above 50%ile) and low within-module degree (below 50%ile) are identified as dynamic integrators, which primarily make allegiances with nodes outside of their reference community. The majority of cerebellar nodes fall into this category. For each quadrant (divided by 50%ile lines), we show the proportion of cortical (left and right) and cerebellar nodes using inset bar plots.

Interestingly, this analysis revealed that all the dynamic hubs were located in the cortical regions, while the cerebellar nodes were overwhelmingly identified as dynamic integrators. Inspection of the summary bar plots in Figure 5, we see that nearly 2 times the proportion of cerebellum nodes, compared with cortical nodes, are likely to be dynamic integrators, as defined by being in the top 50 percentile of participation coefficients and below median within-module degrees. In contrast, no cerebellum node reached dynamic hub properties, being well below the 95th percentile for the within-module degree (as computed on the allegiance matrices).

To further clarify this distinction, we also examined the allegiance changes across the cerebellum and cortex, investigating whether the dynamic reconfigurations induced by the stimulation may be driven by a prioritization of dynamic changes through cortical hubs. Instead, we found that the allegiance changes in the cerebellum were on par with those in the cerebral cortex, suggesting that the dynamic changes are likely being captured solely by the integration and recruitment changes (see Supporting Information Figure S5).

## DISCUSSION

Intrinsic cortical network structure is not static; instead, network structures change dynamically over time during rest and when engaged in cognitive tasks (Telesford et al., 2016) and at rest (Dong et al., 2019; Haller et al., 2013; Rungratsameetaweemana et al., 2022; Yin et al., 2020). Additionally, neuromodulation has the potential to induce prolonged intervals of altered cortical dynamics between functionally connected circuits (Edwards et al., 2019; To et al., 2018). On the basis of previous observations that the cerebellum mediates the structure of cortical dynamics (Georgescu Margarint et al., 2020; Heck et al., 2023; McAfee et al., 2022; Shine, 2021), we hypothesize that its neuromodulation has the potential to induce widespread changes in network structure. In this study, we used network neuroscience tools to characterize the dynamics of a resting-state community structure following a 1-Hz inhibitory stimulation (“perturb and measure”) delivered to the Crus 1 of the lateral cerebellum. We evaluated key metrics of the dynamic network behavior that characterize patterns in community affiliation over time, with emphasis on the movements and distribution of nodes across community boundaries.

The neuromodulation induced significant changes in flexibility and promiscuity among nodal affiliations in both cerebral and cerebellar cortex, indicating that the cerebellar stimulation enhanced intrinsic network flexibility and introduced a period of rapid reconfigurations. Interestingly, we found that the specifics of these changes could be captured by an increase in intercommunity network integration and a decrease in overall intracommunity recruitment. Moreover, cerebellar nodes emerged as dynamic integrators within the brain, in contrast to the dynamic hubs found in cortical regions, which is consistent with the role of the cerebellum as a mechanism for promoting efficiency in cognitive and motor function via the coordination of neural units (Ito, 2008; Koziol et al., 2014; Schmahmann, 2010).

Our findings illustrate the stabilizing nature of the cerebellum on cortical dynamics and the potential for widespread disruption of functional architectures when that integrative function is disturbed. It is important to note that our findings reflect changes in the dynamics of network community configurations, rather than wholesale disinhibition of thalamo-cerebellar cortical pathways, as evaluated here in the resting state. Nonetheless our observations (Supporting Information Figure S6) are consistent with recent theoretical proposals in which the cerebellum is part of a thalamocortical circuit that facilitates segregation among functional networks during tasks associated with well-learned patterns, effectively stabilizing ongoing network dynamics (Heck et al., 2023; Shine, 2021). The distributed impact of rTMS on larger cortical dynamics is also consistent with theories that link maladaptive spreading of dysfunction via integrator nodes in the case of disease (Fornito et al., 2015). Our approach further highlights the nuanced role of network dynamics in understanding the interplay of large-scale functional systems.

### Cerebellar Stimulation Increases Network Flexibility

Flexibility characterizes the likelihood of nodal affiliation changes over time, with studies showing that this metric correlates with improved cognitive function, including faster learning rates (Bassett et al., 2011), increased reinforcement learning of visual cues and outcomes (Gerraty et al., 2018), improved working memory performance (Braun et al., 2015), the need for cognition and creative achievement (He et al., 2019), and the resistance to online influence (Lima Dias Pinto et al., 2022). Promiscuity characterizes the distribution of these reconfigurations across potential unique affiliations, computed as the likelihood of nodes affiliating with every other community at least once.

We found that, on average, 40% of nodes *increased* promiscuity after stimulation (Figure 3B), with cerebellar nodes more likely to exhibit distributed dynamic changes that include affiliations to many other communities. This finding is consistent with the characterization of inhibitory rTMS as inducing a disruption in underlying cortical dynamics (Edwards et al., 2019; Siebner et al., 2022) and with previous studies showing that neuromodulation over the cerebellum propagates to functionally connected circuits in the cerebral cortex (Farzan et al., 2016; Halko et al., 2014; Rastogi et al., 2017). Our results further demonstrate substantial and global cortical response to cerebellar stimulation. Importantly, nearly all of the nodes that changed in flexibility exhibited an *increase* in flexibility (68%). Together with the studies above showing improvements in cognitive function associated with increased flexibility, our results further suggest that cerebellar rTMS may have the potential to induce functional plasticity into network architectures that allow for meaningful dynamic reconfigurations of community structure, rather than being purely disruptive.

At first blush and considering our findings, one may propose cerebellar stimulation as an intervention technique that may be harnessed and deployed for improved behavioral outcomes (e.g., enhanced working memory); however, as Safron et al. (2022) point out, while elevated flexibility may be associated with adaptive behaviors, excessive flexibility could also reflect deleterious cognitive outcomes (Betzel et al., 2017) and could also be indicative of pathology (e.g., Braun et al., 2016). For example, within development, excessive flexibility in the visual areas of infants was negatively correlated with the rate of developmental milestone achievement (Yin et al., 2020), whereas increased flexibility in somatomotor areas and higher-order brain regions was typical during developmental progression, indicating a role for flexibility in adapting to novel situations and challenges.

It is important to note that the stimulation protocol employed in this study was low-frequency repetitive stimulation, an inhibitory protocol. Behavioral outcomes using similar inhibitory protocols over the cerebellum have translated into poorer task performance, illustrated by an increase in error rates in a behavioral inhibition task (Esterman et al., 2017) and a decrease in sensory responses to auditory stimuli (Andrew, 2020). Additionally, previous studies utilizing cortical stimulation have documented relatively modest changes in flexibility following visual and parietal stimulation (Garcia, Ashourvan, et al., 2020) with equally modest behavioral changes (Garcia, Battelli, et al., 2020). Together, these results indicate the need for further research to evaluate the interaction between stimulation protocols and sites, and the potential to induce optimal levels of cortical network flexibility for particular tasks. The relatively large and nonspecific changes in flexibility (and promiscuity) observed here in the resting state should be taken as evidence for the potential of cerebellar stimulation to elicit *excessive flexibility* in the cortex, with future research evaluating the specificity of behavioral outcomes.

### Cerebellar Nodes Uniquely Act as Integrators Within the Brain

In humans, Crus I and II of the lateral cerebellum are the largest of the lobes and are expanded in humans and apes relative to other mammals (Buckner, 2013; Habas, 2021; Strick et al., 2009). Resting-state connectivity reveals these lobes to be nestled between and distinct from somatomotor maps in the cerebellum, and highly connected to prefrontal and parietal cortices (Buckner et al., 2011; Krienen & Buckner, 2009). Moreover, graphical analysis of the cerebellum in the resting state reveals its functional connections to have small-world properties, meaning that the architecture of the graphs strikes a balance between regular connectedness and fully random (Pezoulas et al., 2017), with significant individual differences in the small worldness and hierarchy (Chen et al., 2022).

The interpretation that the cerebellar nodes in our study were characterized by their high participation coefficients and lower within-module degree suggests a propensity toward integrating information across different brain regions. This is consistent with massive expansion of input representations in the cerebellum, a key feature that promotes multifunctionality despite the highly uniform cytoarchitecture (Andersen et al., 1992; Diedrichsen et al., 2019; Herculano-Houzel, 2010). Moreover, cerebellar Purkinje cells possess significantly more dendritic spines than typical cortical cells, allowing for the broad integration of inputs at a speed and scale beyond the operating parameters of the cerebral cortex, providing a computational vehicle to rapidly integrate cortical information (Walter & Khodakhah, 2009).

It is also important to consider the broader role of the cerebellum in regulating cortical network dynamics. Recent theoretical models position the cerebellum as part of a larger thalamocortical circuit that promotes segregation in ongoing network dynamics supporting distinct functions (Heck et al., 2023; Shine, 2021). These models emphasize how, in the baseline state, tonically active Purkinje cells release inhibitory Gamma-Aminobutyric Acid (GABA) onto deep cerebellar nuclei that project to thalamic targets. This inhibition constrains the dynamics of the larger feedforward thalamocortical network and, when released, provides a mechanism by which well-learned patterns can constrain network states. Interpreted within this theoretical framework, inhibitory rTMS (such as the protocol used in this study) disrupts this mechanism, releasing the constraint on feedforward thalamocortical circuits and effectively flattening the landscape of possible cortical states. Inhibitory neuromodulation is therefore predicted to promote a shift to more integrative network configurations, consistent with what we observed in the first 20 min poststimulation.

The importance of the cerebellum in constraining neural dynamics is consistent with the emerging understanding that the cerebellum functions, at least in part, to optimize complex cognitive and motor functions (Honda et al., 2018; Koziol et al., 2014). Whereas damage to the cerebellum rarely results in profound cognitive deficits, neuropsychological tests reveal a more subtle but pervasive impact on language, working memory, and executive function (Schmahmann, 2010). In both cognitive and motor function, the cerebellum is believed to support the integration of internal models (motor execution or mentalizing processes) within incoming sensorimotor signals so as to rapidly correct for errors and promote the skillful execution of cognitive and motor acts (Buckner, 2013; Ito, 2008). It should not be surprising, then, that our analyses of intrinsic connectivity reveals highly integrative properties in cerebellar nodes. Because these integrative properties are highly susceptible to neurostimulation, our findings emphasize network roles better described by functional adaptability over structural constraints. Our results also add to a mechanistic understanding of how multifunctionality in the cerebellum may be explained as a balance of integration and segregation across cognitive networks more broadly.

### Cerebellar Nodes’ Nonspecific Integration

Further inspecting the connections of the highly integrative nodes of the cerebellum to the cortex, it may be efficient to utilize the hubs within the cortex to maximally impact cortical functions; however, as shown in a supplementary analysis (Supporting Information Figure S5), our results indicate that the allegiance patterns between nodes within the cortex and the cerebellum are quite nonspecific. In other words, we observe that the cerebellum integrative properties are not driven by clear hub communication to the cortex; instead, its connections are sparse and distributed across the brain. This is similar to the concept of a *diverse club*, where nodes and edges are critical for efficient global communication (Bertolero et al., 2017). Our dynamic augmentation to the diverse club concept allows us to expand upon this construct, suggesting that dynamic diverse clubs within the brain may drive global communication and impact cognition in a general, nonspecific fashion. In other words, by not focusing solely on cortical hubs, the cerebellum might gain a more comprehensive understanding of the overall *state* of the cortex, rather than simple read-out of dynamic hubs within cortex, allowing the integration of *contextual* information from many diffuse and functionally diverse cortical areas. Such an integrative role might enable the cerebellum to better predict and respond to the dynamic demands of the brain, maintaining a seamless coordination between different functional domains, similar to the previous findings that suggest a tight coupling of forward modeling and state estimation for movement (e.g., Hwang & Shadmehr, 2005). This capacity for extensive contextual integration suggests that the cerebellum is not merely a passive recipient of cortical inputs but an active participant in shaping the brain’s functional architecture.

### Rapid and Widespread Dynamic Whole-Brain Reconfigurations

In addition to the cerebellum specific results, our analyses using dynamic community detection revealed a time-evolving network connectivity with an average of five communities identified within an individual for any given time window. When comparing these networks to the prestimulation condition baseline, we observed highly variable community structures across participants, indicating a broad and dynamic architecture. Specifically, the consensus community estimation across subjects and time indicated poor alignment with standardized functional networks, such as the default mode network and control network, with normalized mutual information (NMI) values never exceeding a moderate level of consistency (NMI < 0.32). This variability and lack of alignment highlight the brain’s inherent elasticity and dynamic propensity (Safron et al., 2022) and is consistent with observations that network structure in the cerebellum is highly individual (Marek et al., 2022).

The observed poor alignment across individuals suggests that each individual’s intrinsic network configuration is unique in its dynamic reconfigurations, underscoring the importance of treating the brain as a highly dynamic system. It also implies that personalized approaches may be necessary for understanding and effectively targeting brain function and disorders, rather than relying on a one-size-fits-all model based on group averages (Dubois & Adolphs, 2016). The brain’s capacity for such extensive reconfiguration may be fundamental to its adaptability and resilience, allowing for a flexible response to ever-changing environmental demands.

Finally, it is worth noting that alignment of the cerebellum is more challenging than the cerebral cortex, due in part to the relatively smaller lobule structure, the tight folding of the lobes, and the weighting of large-scale cortical features in standard volumetric normalization procedures (for a review, see Schlerf et al., 2014). A strength of computing integration and recruitment using each individual’s intrinsic network configuration is to minimize the impact of interindividual misalignments on subsequent network metrics. Normalization approaches tailored specifically for the cerebellum, such as SUIT (Diedrichsen, 2006), coupled with cerebellar-specific probabilistic atlas templates, have the potential to minimize variability due to misalignments in template normalization.

### Limitations and Considerations for Future Research

It is important to note some limitations of this study. One of which is the strength of causal inference given that a sham condition or control site is not included. This leaves open the potential for the observed effects to reflect, in part, secondary effects such as discomfort, disorientation, and fatigue. We note, however, that the subjects reported no discomfort or fatigue, which is consistent with other studies that have used the butterfly coil to deliver 1-Hz cerebellar TMS for repetitive stimulation combined with neuroimaging (Cho et al., 2012; Fierro et al., 2007). This is in contrast to the double cone coil, the first to effectively target the cerebellum, which is reported as more uncomfortable than the butterfly coil (i.e., Fernandez et al., 2018). We also note that combined sequential rTMS and fMRI in prior work with healthy subjects indicate fatigue is not a major confounding factor, even in stimulation protocols with protocols more extended than ours. For example, Richter et al. (2013) examined repeated motor threshold measurements using a figure-8 coil across 1.5-hr TMS sessions and reported minimal fatigue effects even under these extended conditions. This finding suggests that session durations of approximately 20 min, as in the present study, are unlikely to induce significant fatigue in most participants.

## CONCLUSION

Our findings suggest that the cerebellum, through its integrative properties and sparse, distributed connections to the cortex plays a unique role in modulating brain network dynamics. Unlike the cortical regions that act as dynamic hubs, the cerebellum emerges as a dynamic integrator, facilitating the rapid and flexible reconfiguration of brain networks. This broad connectivity pattern may provide the cerebellum with a more comprehensive understanding of the cortical state, integrating rich contextual information that enhances its ability to modulate cognitive and motor functions. This mechanism of integrative capacity is consistent with dysmetria of thought, where cerebellar disruption can impair higher-order behavior by affecting the cerebellum’s modulation of cognitive operations. This perspective could suggest avenues for future research, exploring the cerebellum’s contributions to brain function, perhaps encompassing a fundamental role in cognitive integration and adaptability (Honda et al., 2018). The brain’s inherent plasticity and dynamic nature, highlighted by our study, further reinforce the need to treat it as a flexible system, capable of extensive reorganization in response to varying demands, and underscore the potential for personalized approaches in neuroscience research.

## Acknowledgments

This research was supported by the U.S. Army DEVCOM Army Research Laboratory through mission funding (J. O. G.), army educational outreach program (K. B., W911SR-15-2-0001), and the Italian Ministry of University and Research (Z. C.; PRIN 20203LT7H3). The views and conclusions contained in this document are those of the authors and should not be interpreted as representing the official policies, either expressed or implied, of the U.S. DEVCOM Army Research Laboratory or the U.S. Government. The U.S. Government is authorized to reproduce and distribute reprints for Government purposes notwithstanding any copyright notation herein.

## Supporting Information

Supporting information for this article is available at https://doi.org/10.1162/NETN.a.541.

## Author Contributions

Kanika Bansal: Conceptualization; Formal analysis; Methodology; Software; Visualization; Writing – original draft; Writing – review & editing. Zaira Cattaneo: Funding acquisition; Investigation; Writing – review & editing. Viola Oldrati: Investigation; Writing – review & editing. Chiara Ferrari: Investigation; Writing – review & editing. Emily D. Grossman: Data curation; Writing – original draft; Writing – review & editing. Javier O. Garcia: Conceptualization; Funding acquisition; Methodology; Software; Visualization; Writing – original draft; Writing – review & editing.

## Funding Information

Kanika Bansal, DEVCOM Army Research Laboratory (https://dx.doi.org/10.13039/100019923), Award ID: W911SR-15-2-0001. Zaira Cattaneo, Italian Ministry of University and Research, Award ID: PRIN 20203LT7H3.

## Supplementary Material



## TECHNICAL TERMS

Community structure: The overall organization of modules within a network.
Inhibitory repetitive transcranial magnetic stimulation: Noninvasive brain stimulation protocol that uses trains of low-frequency magnetic pulses to temporarily decrease excitability of the underlying neural tissue and functionally connected neural populations.
Wavelet coherence: A method to measure time-varying correlations between two signals across different frequency bands.
Network reconfigurations: Dynamic changes in how brain regions connect and cluster over time.
Null model: A baseline randomized model used for statistical comparison.
Bootstrapping: A resampling technique to estimate reliability and/or variability of results.
Cohen’s *d*: A standardized measure of effect size to quantify the difference between two groups means.
Resting-state connectivity: Spontaneous, low-frequency correlations in neural activity between brain regions apparent when the participant is not engaged in a task, revealing intrinsic functional architecture.
Integration and segregation: Balancing network-wide communication (integration) with specialized, localized processing (segregation).
Network modules: Groups of highly connected brain regions or nodes with higher within-group connectivity than between-group connectivity.
