# Supplementary material for: Intrinsic brain network dynamics modulated by neural stimulation to cerebellum
Source: Netw Neurosci. 2026 Apr 22;10(2):329–51. doi: 10.1162/NETN.a.541 (PMC13108502; doi:10.1162/NETN.a.541)
Supplement: Supplementary file 1 [file netn-10-2-329-s001.pdf]

Supplementary Material

Intrinsic brain network dynamics modulated by neural stimulation to cerebellum

Kanika Bansal[1,2,*], Zaira Cattaneo[3], Viola Oldrati[4], Chiara Ferrari[5,6], Emily D. Grossman[7], Javier O. Garcia[1,*]

[1]Humans in Complex Systems Division, U.S. Army DEVCOM Army Research Laboratory, Aberdeen Proving Ground, MD 21005 USA

[2]Department of Computer Science and Electrical Engineering, University of Maryland, Baltimore County, MD USA

[3]Department of Human and Social Sciences, University of Bergamo, Italy

[4]Scientific Institute, IRCCS E. Medea, Bosisio Parini (LC) 23842, Italy

[5]IRCCS Mondino Foundation, Pavia 27100, Italy

[6]Department of Humanitas, University of Pavia, Pavia 27100, Italy

[7]Department of Cognitive Sciences, University of California, Irvine, Irvine, CA USA

*Corresponding authors' emails: phy.kanika@gmail.com; javiomargarcia@gmail.com

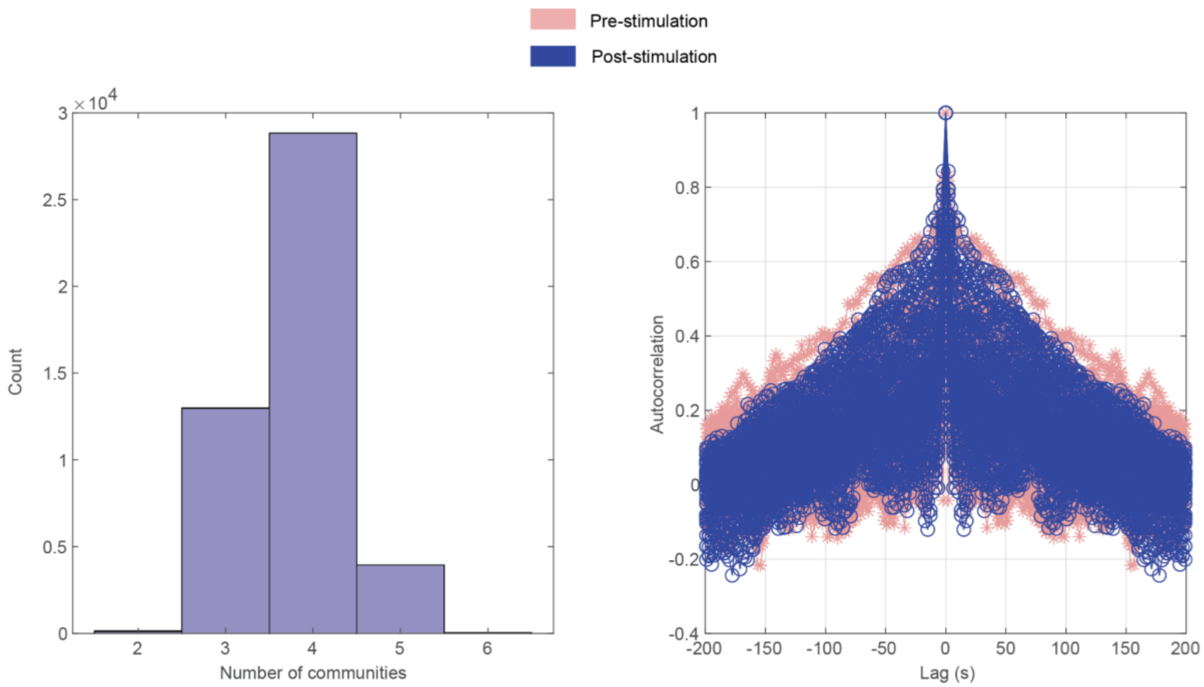

Figure S1: Left: Histogram of the number of communities across time points (and iterations), aggregated over all subjects, shown separately for pre-stimulation and post-stimulation conditions. Pre- and post-stimulation histograms are largely overlapping (shown as purple). Right: Autocorrelation of fMRI signals from different brain regions for a representative subject, pre- and post-stimulation. These plots again demonstrate very strong similarity between pre- and post-stimulation data, confirming that stimulation did not introduce structural changes in the time series.

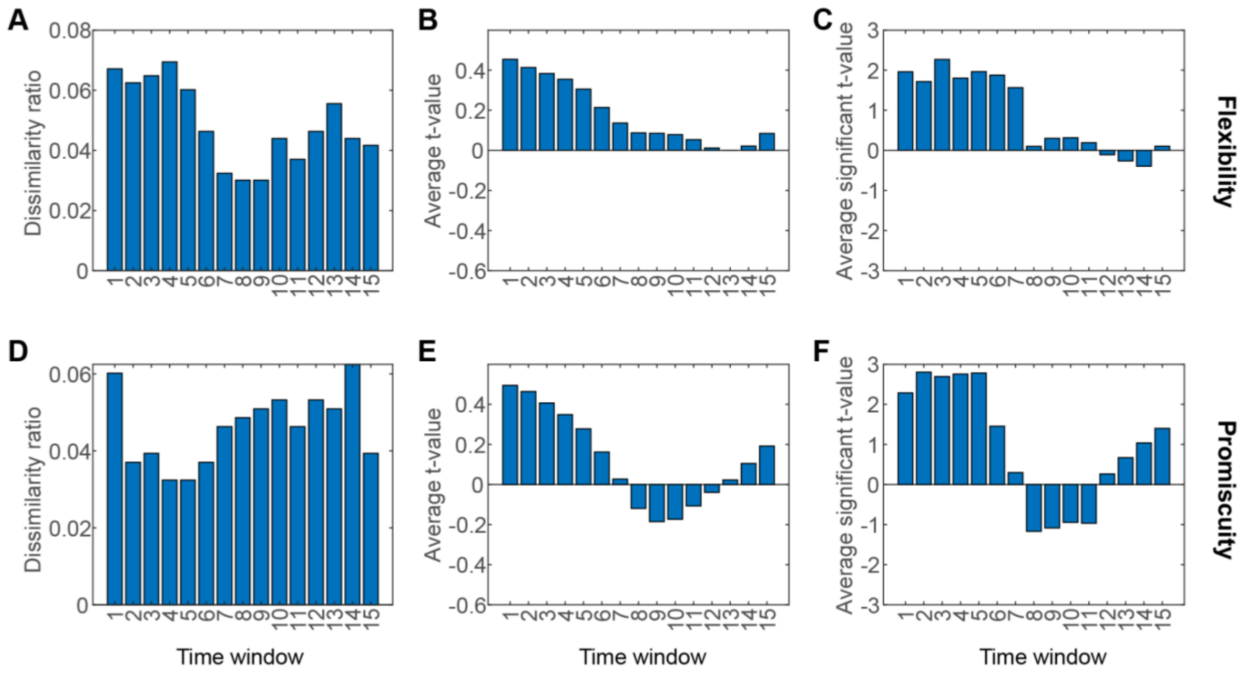

Figure S2: Comparing post-stimulation and pre-stimulation conditions as a function of time. We used sliding time windows with 67% overlap. Each time window here represents an average of three consecutive time points for which metrics of interest were calculated (e.g., in Figure 2B-E). (A),(D) Dissimilarity ratio as a function of time for flexibility and promiscuity respectively. (B),(E) Average t-value as a function of time across brain nodes when flexibility and promiscuity were compared post-stimulation as compared to pre-stimulation. High positive values indicate strong increase in the metrics post-stimulation across many nodes. Values close to zero indicate either low t-values across all nodes or symmetrically distributed positive and negative t-values, likely indicating the fading impact of stimulation. (C),(F) Average t-value only for nodes that showed significantly different (p-value < 0.05, uncorrected) flexibility and promiscuity respectively, post-stimulation compared to pre-stimulation.

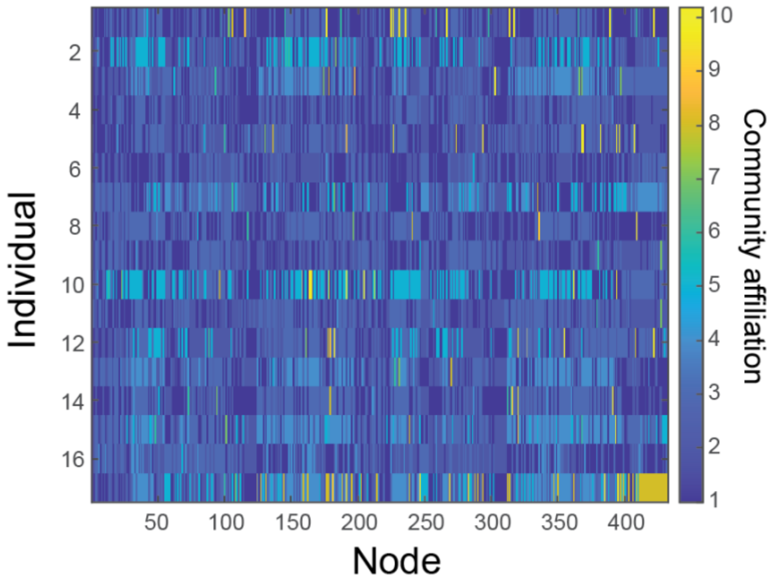

Figure S3: Individual consensus community. We obtained a consensus community structure for each individual by first calculating a consensus similarity across all the temporal windows for which community detection was performed (i.e., 28 windows) and then calculating consensus iterative for all the iterations (i.e., 100 iterations) of dynamic community detection. Consensus similarity estimates a single representative partition from a set of partitions (here 28) that is the most similar to all others. Consensus iterative identifies a single representative partition from a set of partitions (here 100), based on statistical testing in comparison to a null model. Occasionally, in consensus community structure we observed communities with single nodes, which were excluded from further analysis. Most of the nodes were distributed between 6 or fewer communities in any given subject.

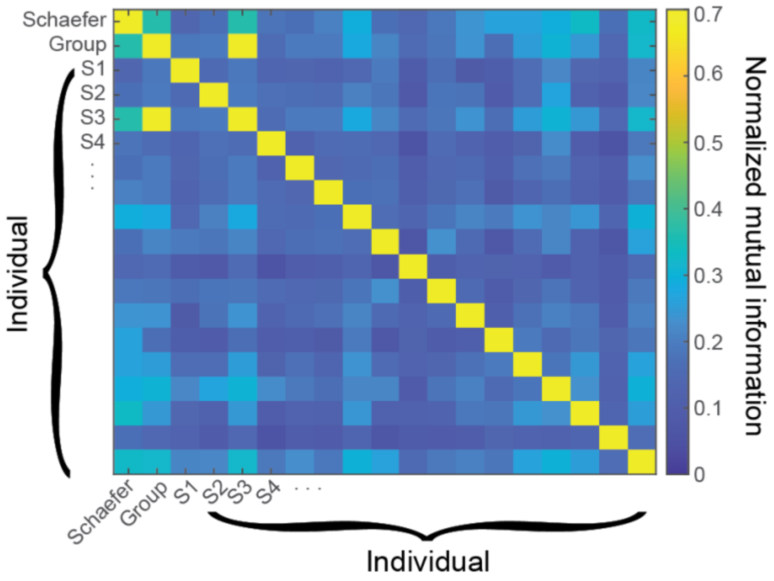

Figure S4: Comparison between different community partitions using normalized mutual information (NMI) between different community structures. NMI is a measure of similarity between two different partitions of community structure and is bounded between 0 and 1. We used two different functional characterizations of *standardized networks.* The first is a data driven community structure that is completed by estimating the consensus similarity between individual consensus communities (from Figure S3), which found 10 communities across subjects ("Group"), and another using the functional atlas labels provided by the chosen parcellation, which used labels consistent with previously understood functional networks (e.g., Default Mode Network, Control Network, Limbic System, etc.) as part of the Schaefer parcellation. We estimated NMI between communities, comparing each subject to one another in addition to the *Group* network and *Schaefer* network compositions. Neither consensus community *Group* network nor *Schaefer* network compositions substantially deviated from the inter-subject NMI, where the average NMI on inter-subject NMI was M = 0.16 (SD = 0.03), and that of the Group was 0.21 and Schaeffer was 0.23. In fact, NMI across all comparisons never exceeded 0.32, which indicates a poor alignment of overall network architecture across individuals.

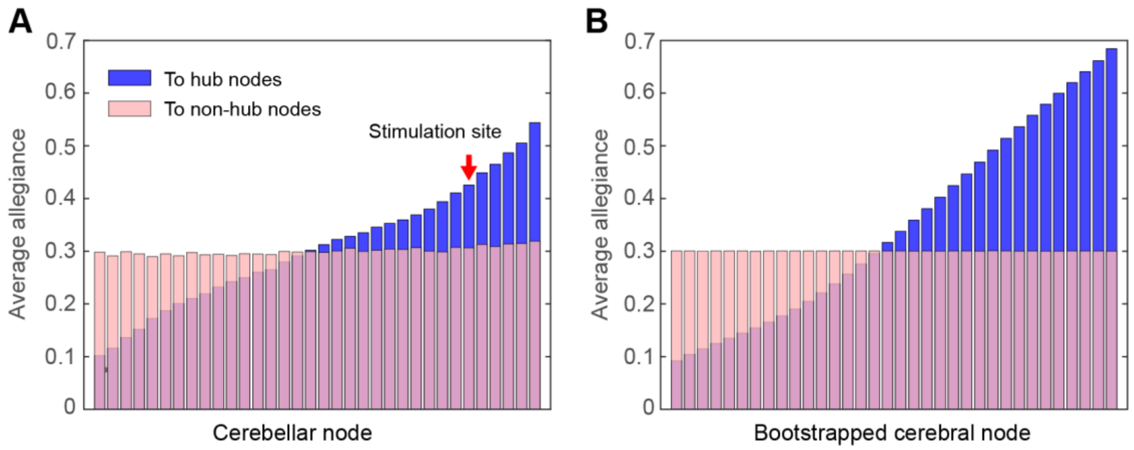

Figure S5: (A) Average allegiance of cerebellar nodes with hubs and non-hubs in the cerebral cortex. Hubs are defined as nodes with high overall allegiance (95%ile). Nodes are arranged in the order of increasing average allegiance to hub nodes. (B) Same as A, except that the equal number of nodes as cerebellum, i.e., 34, were picked within the cerebral cortex using a bootstrapping process 1000 times. Average allegiance values are computed by taking average across those bootstrapping iterations. Similar connectivity profiles for cerebellar and cerebral nodes are evident.

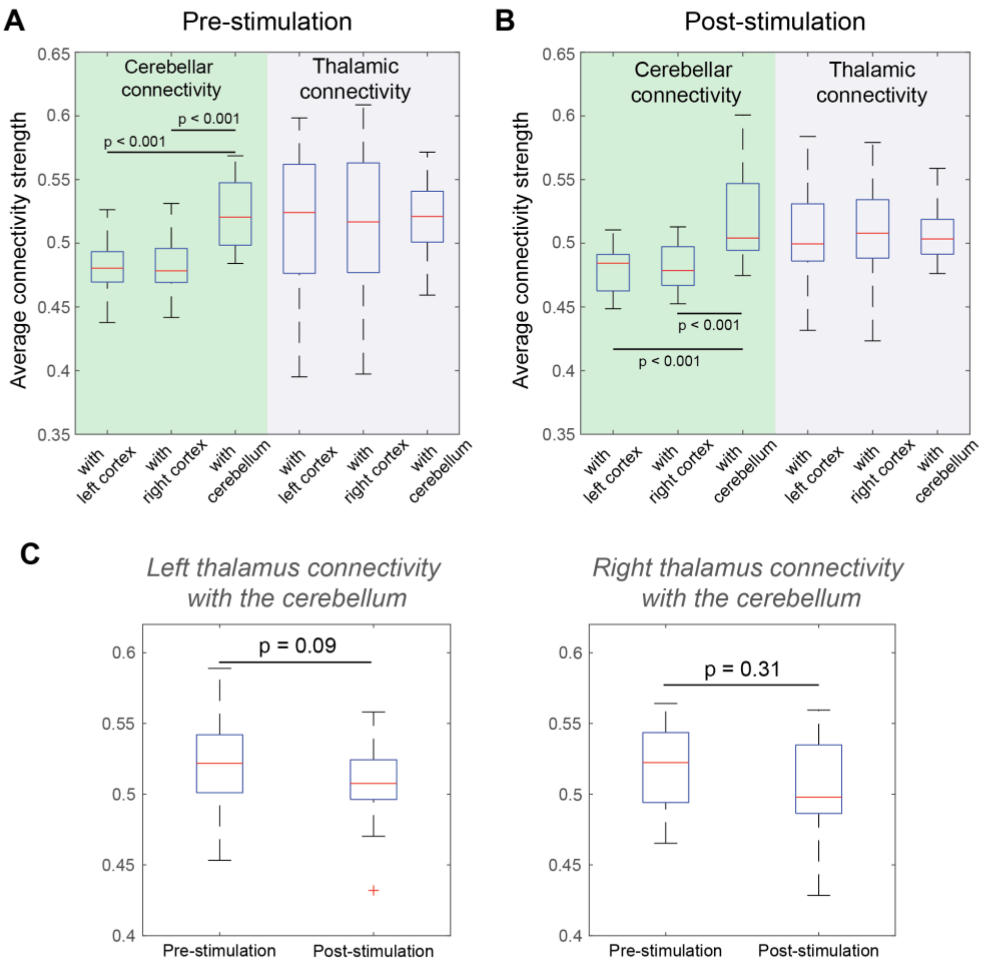

Figure S6: (A) and (B) compare connectivity (coherence) of the cerebellar and thalamic nodes (left and right) with the cortex and the cerebellum (A) pre- and (B) post-stimulation (using the same temporal window as the main paper). The cerebellum exhibited high within-structure connectivity but significantly lower connectivity with the cortex in both conditions (t-test, $p < 0.001$, df = 15). In contrast, the thalamus showed strong connectivity with both the cortex and the cerebellum, consistent with its proposed role as a critical mediator in the cerebello-thalamo-cortical pathway (Shine, 2021; Progress in Neurobiology 199, 101951). This connectivity pattern aligns with theoretical models suggesting that cerebellar influence on cortical dynamics is primarily routed through thalamic regions. (C) Direct comparison of stimulation effects on thalamus-cerebellum connectivity showed only a marginal change, limited to the left thalamus

connectivity (t-test, $p = 0.09$, $df = 15$). This finding may reflect the complex nature of cerebellar neuromodulation, which likely affects both excitatory and inhibitory thalamocortical projections (Fernandez et al., 2020; The Cerebellum, 19(2), 309–335), and suggests that task-based paradigms may be necessary to fully reveal modulatory effects on this pathway (Grimaldi et al., 2014; The Cerebellum, 13(1), 121–138). While these results do not definitively establish thalamic mediation, they support the connectivity patterns predicted by theoretical models, though task-based paradigms may be necessary to reveal causal mechanisms.
